# Theoretical Study on the Gas Phase and Gas–Liquid Interface Reaction Mechanism of Criegee Intermediates with Glycolic Acid Sulfate

**DOI:** 10.3390/ijms24043355

**Published:** 2023-02-08

**Authors:** Lei Li, Qingzhu Zhang, Yuanyuan Wei, Qiao Wang, Wenxing Wang

**Affiliations:** Environment Research Institute, Shandong University, Qingdao 266237, China

**Keywords:** Criegee intermediates, glycolic acid sulfate, gas-phase reaction, aqueous-surface reaction, proton transfer

## Abstract

Criegee intermediates (CIs) are important zwitterionic oxidants in the atmosphere, which affect the budget of OH radicals, amines, alcohols, organic/inorganic acids, etc. In this study, quantum chemical calculation and Born–Oppenheimer molecular dynamic (BOMD) simulation were performed to show the reaction mechanisms of C2 CIs with glycolic acid sulfate (GAS) at the gas-phase and gas–liquid interface, respectively. The results indicate that CIs can react with COOH and OSO_3_H groups of GAS and generate hydroperoxide products. Intramolecular proton transfer reactions occurred in the simulations. Moreover, GAS acts as a proton donor and participates in the hydration of CIs, during which the intramolecular proton transfer also occurs. As GAS widely exists in atmospheric particulate matter, the reaction with GAS is one of the sink pathways of CIs in areas polluted by particulate matter.

## 1. Introduction

The reactions of ozone with unsaturated hydrocarbons are important sources of free radicals and particulate matter in the atmosphere [1]. Criegee intermediates (CIs) are tropospheric biradical/zwitterionic species that are derived from the ozonolysis of alkenes [2]. Ozone undergoes 1,3-cycloaddition with a double bond of alkene to form a primary ozonide that subsequently decomposes into a carbonyl oxide (also called Criegee intermediate) and a carbonyl compound [3]. The energized Criegee intermediate, which is produced by exothermic decomposition and contains vibrational excitation, undergoes unimolecular decay or forms a stable Criegee intermediate via collisional quenching [4]. In addition to the above reaction, the contribution that formed through other reactions to the total CIs is inappreciable. The reaction of CH_3_O_2_ with OH radicals, affecting the concentrations of HO_2_ and O_3_ in the oceanic boundary layer, has been determined to produce trace amounts of CH_2_OO (yield of CH_2_OO < 5%) [5]. The oxidation of dimethyl sulfoxide forms CH_2_OO at low temperatures, but the yield is too low to be a pivotal source of CIs [6].

The unimolecular decomposition of CIs, which occurs via the vinyl hydroperoxide pathway or the ester pathway, plays a significant role in the production of atmospheric OH radicals [7,8]. Elshorbany et al. [9] reported that the decomposition of CIs has a 24% contribution to the daytime OH radical formation. In addition, CI decomposition is a steady and dominant source of OH radicals at nighttime [10]. The PUMA campaign (a project that measured pollution of the urban midlands atmosphere in the UK) showed that ozonolysis of alkenes dominates the OH production in winter and accounts for more than 50% of the production in summer [11].

Even if most CIs decay through unimolecular pathways, a fraction of CIs survives for long enough to react with other substances. The reaction with water vapor is the most efficient scavenging route for CIs in the troposphere [12]. Part of CIs react with water molecules and form low vapor pressure substances that act as cloud condensation nuclei (CCN) [13]. CH_2_OO reacts with NO_2_ to enhance NO_3_ production in the urban atmosphere [14]. Dimethyl-substituted Criegee intermediate (CH_3_)_2_COO survives at high humidity to react with atmospheric SO_2,_ and the reaction rate (1.3 × 10^−10^ cm^3^s^−1^) is close to the gas kinetic limit [15]. The reactions of CIs with atmospheric organic matter play a significant role in the formation of secondary organic aerosol (SOA). The product of the CH_2_OO + acrolein reaction is the secondary ozonide that has the potential to form SOA because of its large molecular weight [16]. Experimental and theoretical studies show that the reaction of CIs with alcohols represents a considerable source of α-alkoxyalkyl hydroperoxides (AAAHs) that form SOA with a production rate of 24 Gg year^−1^ in tropical forests [17]. Despite the fact that the reactions of CIs with organics have been internationally recognized, the understanding of a Criegee–organosulfate reaction in the atmosphere is still developing.

Organosulfates (OSs), also known as sulfate esters or sulfate derivatives, are important organosulfur compounds that contain -OSO_3_H group in the atmosphere [18,19]. Due to hydrophobic (the hydrocarbon group) and hydrophilic (the sulfate group) parts, OSs lower the surface tension of atmospheric particles, eventually changing the ability of particles to uptake water and to form CCN [20,21]. As OSs absorb sunlight, they significantly change the optical properties of organic aerosols and affect the energy balance of the atmosphere [22]. OSs react with oxidants such as OH, O_3_, and NO_X_ in the atmospheric lifetime, which promotes the formation of SOA [23]. Accordingly, OSs have the potential to react with CIs. In the atmosphere, glycolic acid sulfate (GAS) is a common organosulfate with a -OSO_3_H group and a -COOH group [24]. Some studies have reported the formation of aqueous-phase and heterogeneous OSs [25,26]. Most of the GAS is present in the ambient aerosols in the atmosphere [27]. Detection of OSs in summer in Beijing revealed that GAS is the most abundant among all quantified species, the concentrations of GAS in the thirteen OSs measured ambient aerosols ranged from 3.9 to 58.2 ng/m^3^ and the average concentration was 19.5 ng/m^3^ [28]. Olson et al. measured concentrations of OSs in particulate matter in the United States, Pakistan, and Mexico, where GAS concentrations ranged from 1.9–11.3 ng/m^3^ [29]. Therefore, it is necessary to investigate the reaction mechanism of CIs with GAS in the atmosphere.

This work explored the reaction mechanism of C2 CIs (*anti*- and *syn*-CH_3_CHOO) with GAS using density functional theory (DFT). Due to the hydrophobic methyl group, C2 CIs have a longer lifetime at the gas–liquid interface compared to C1 CIs [30]. Therefore, the Born–Oppenheimer molecular dynamics (BOMD) was carried out to simulate the heterogeneous reactions of CH_3_CHOO with GAS on the droplet surface. For the gas–liquid interface reactions, we consider the collision of GAS molecules in the atmosphere with CIs on the droplet rather than the collision reaction of CIs with GAS that is dissolved in the droplet.

## 2. Results and Discussion

**Figure 1:**
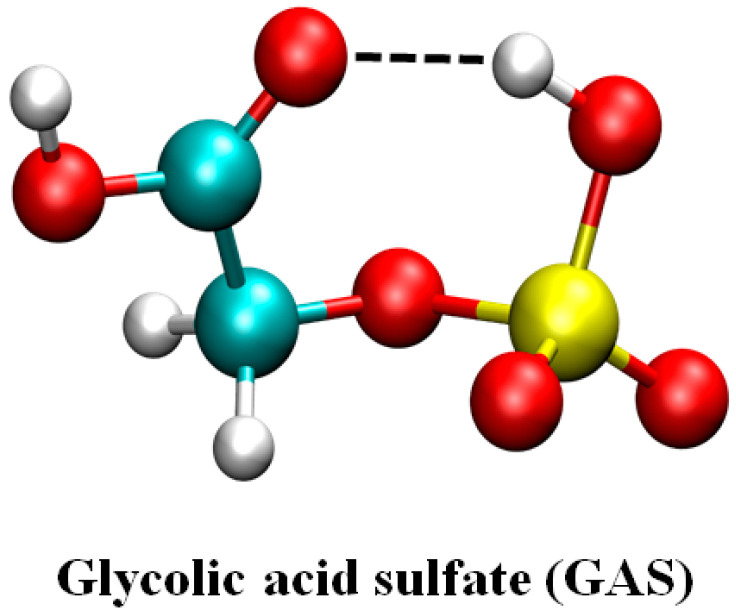
The most stable configuration of GAS.

The most stable configuration of GAS is shown in Figure 1. A seven-membered ring structure is formed in the molecule. The hydrogen bond interaction was observed between the terminal hydrogen of the OSO_3_H group and the carbonyl oxygen of the COOH group.

### 2.1. Gas-Phase Reactions

**Figure 2:**
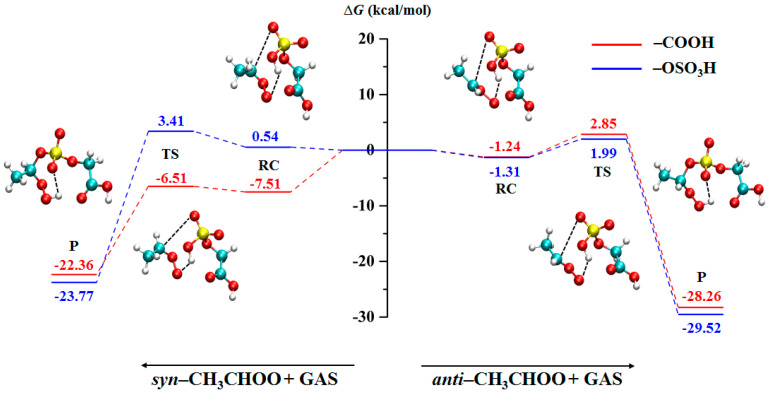
The minimum energy pathway of the direct reactions between CH_3_CHOO and GAS.

The values of relative Gibbs free energy (CCSD/6-311++G(2d,2p)) of the reactions between CH_3_CHOO and GAS are displayed in Figure 2. The configurations of reaction complexes, transition states, and products are depicted in Figure S1. The reaction energy barriers of *syn*-CH_3_CHOO with OSO_3_H and COOH groups are 1.00 and 2.87 kcal/mol, respectively. The reactions of *anti*-CH_3_CHOO with OSO_3_H and COOH groups need to cross energy barriers of 3.30 and 4.09 kcal/mol, respectively. The low energy barriers suggest that these reactions are feasible in the atmosphere. Previous studies have shown that most reactions of CIs with carboxylic acids follow a barrierless pathway [31,32]. However, we observed that the reactions of the COOH group with CH_3_CHOO require overcoming the energy barrier, which may be due to the formation of a six-membered ring that increases the stability of GAS.

**Figure 3:**
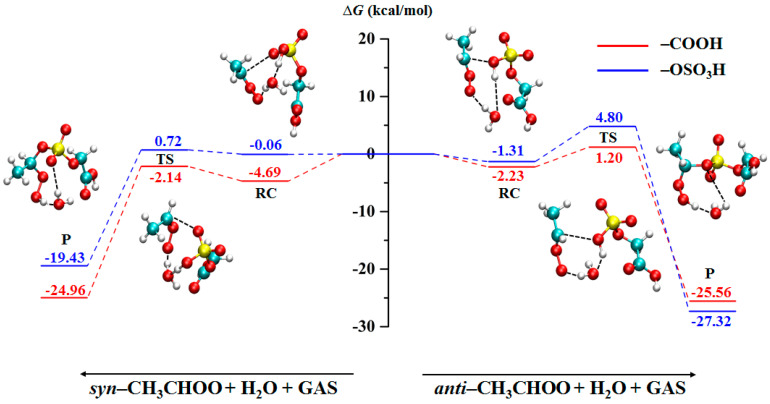
The minimum energy pathway of the water-mediated reactions between CH_3_CHOO and GAS.

Water molecules are one of the most abundant species in the atmosphere and have a significant impact on atmospheric chemical processes [33]. Therefore, the relative Gibbs free energy (CCSD/6-311++G(2d,2p)) of the water-mediated reactions between CH_3_CHOO and GAS was calculated (Figure 3). The two lowest energy barriers are 0.78 and 2.55 kcal/mol, which are determined for the water-mediated reaction of *syn*-CH_3_CHOO with the OSO_3_H group and the reaction of *syn*-CH_3_CHOO with the COOH group, respectively. Compared with the direct reactions of *syn*-CH_3_CHOO, the water molecule lowers the energy barrier of the reaction with the OSO_3_H group but increases the energy barrier of the reaction with the COOH group. In contrast, the water-mediated reaction barrier of *anti*-CH_3_CHOO with OSO_3_H group is increased to 6.11 kcal/mol, and that of *anti*-CH_3_CHOO with COOH group is decreased to 3.43 kcal/mol. The participation of water molecules leads to the change of the reaction energy barrier to be less than 3 kcal/mol, indicating that the effect of water molecules on the reaction of CH_3_CHOO with GAS is weak. The configurations of the water-mediated reaction stages are presented in Figure S2.

### 2.2. Gas–Liquid Interface Reactions

The gas–liquid interface plays a pivotal and ubiquitous role in atmospheric chemistry, including absorbing various pollutants and changing chemical reaction mechanisms. For example, Shang et al. [34] reported the interfacial reaction of SO_2_ with oleic acid, which is a new pathway to form organosulfur in the atmosphere. In this study, the gas–liquid interface reaction mechanism of *anti*-CH_3_CHOO with GAS was explored at the molecular level. In order to eliminate the effect of reaction location, thirty simulations were performed at different locations of the droplet. The reactions of *anti*-CH_3_CHOO with GAS occurred in 16 simulations. The direct and water-mediated reactions of *anti*-CH_3_CHOO with the COOH group occurred six and two times, respectively. The direct and water-mediated reactions of *anti*-CH_3_CHOO with the OSO_3_H group both occurred four times. The hydration reactions of *anti*-CH_3_CHOO occurred 11 times.

#### 2.2.1. Reaction of Anti-CH_3_CHOO with the COOH Group of GAS

**Figure 4:**
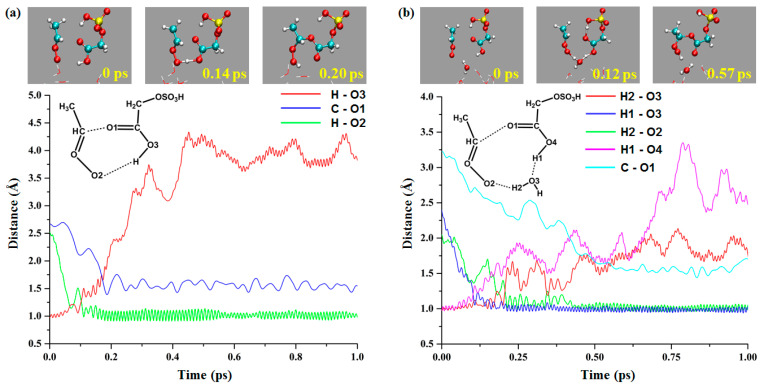
The direct and water-mediated reactions between *anti*-CH_3_CHOO and COOH group of GAS at gas–liquid interface ((**a**): direct reaction; (**b**): water-mediated reaction).

Figure 4a shows the structure and bond length variations of the direct reaction between *anti*-CH_3_CHOO and the COOH group of GAS. Based on the properties of hydrophobicity and hydrophilicity, the methyl group of *anti*-CH_3_CHOO is placed on the side away from the droplet. At 0 ps, the distances of H-O3, C-O1, and H-O2 are 0.97, 2.66, and 2.52 Å, respectively. Subsequently, the H atom of the COOH group gradually approaches the terminal oxygen of *anti*-CH_3_CHOO, and the carbonyl oxygen of the COOH group approaches the α-carbon atom of *anti*-CH_3_CHOO. At 0.14 ps, the distances of H-O3, C-O1, and H-O2 are 1.53, 2.13, and 1.00 Å, respectively, where the transition-state-like structure is formed. At 0.20 ps, the H atom on the COOH group binds to the terminal oxygen of *anti*-CH_3_CHOO, and the C-O1 and H-O2 bonds are formed and remain stable, indicating the formation of the reaction product.

The mechanism of the water-mediated reaction between the *anti*-CH_3_CHOO and COOH group of GAS (Figure 4b) is different from that of the direct reaction. At 0 ps, the initial distances of H2-O3, H1-O3, H2-O2, H1-O4, and C-O1 are 0.98, 2.40, 2.05, 0.97, and 3.22 Å, respectively. At 0.12 ps, the structure of the reactants is similar to the transition state; the distances of H2-O3, H1-O3, H2-O2, H1-O4, and C-O1 are 1.05, 1.22, 1.48, 1.24, and 2.66 Å, respectively. At 0.24 ps, H2-O2 and H1-O4 bonds are formed. However, the C-O1 bond (1.56 Å) is formed at 0.57 ps. In water-mediated reactions, the C-O1 bond is formed later than other bonds, and the water molecule is the bridge of proton transfer.

#### 2.2.2. Reaction of Anti-CH_3_CHOO with the OSO_3_H Group of GAS

**Figure 5:**
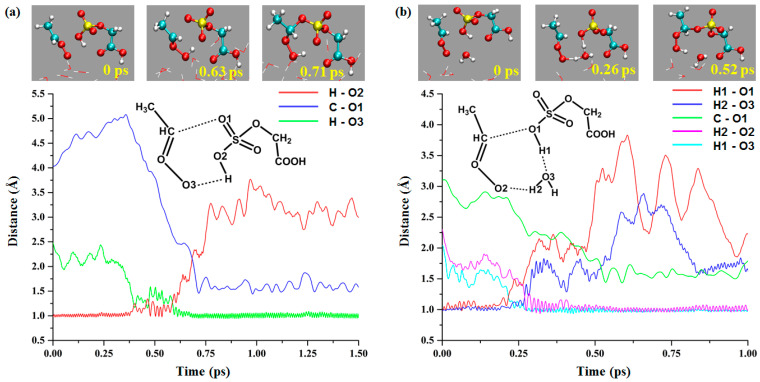
The direct and water-mediated reactions between *anti*-CH_3_CHOO and OSO_3_H group of GAS at gas–liquid interface ((**a**): direct reaction; (**b**): water-mediated reaction).

The direct and water-mediated reactions between the *anti*-CH_3_CHOO and OSO_3_H groups of GAS were observed in our simulations (Figure 5). For the direct reaction, the distances of H-O2, H-O3, and C-O1 are 0.98, 2.48, and 4.04 Å, respectively, at 0 ps. From 0.38 to 0.63 ps, the H atom of the OSO_3_H group vibrates between O3 and O2 atoms. The length of H-O3 fluctuates around 0.98 Å from 0.63 ps, but the distance of C-O1 is still decreasing. At 0.71ps, the O1 atom of GAS binds to the C atom of *anti*-CH_3_CHOO, indicating the formation of a hydroperoxide product.

For the water-mediated reaction (Figure 5b), the initial distances of H2-O2, H1-O3, and C-O1 are 2.30, 2.03, and 3.10 Å, respectively. The transition-state-like structure is observed at 0.26 ps, where the lengths of H2-O2, H1-O3, H1-O1, H2-O3, and C-O1 are 1.41, 1.09, 1.39, 1.10, and 2.43 Å, respectively. The H2-O2 and H1-O3 are formed at 0.30 and 0.27 ps, respectively. The O1 atom binds to the C atom at 0.52 ps, which occurs later than the formation of the H2-O2 bond. This phenomenon is also observed in the water-mediated reaction between the COOH group of *anti*-CH_3_CHOO and GAS, suggesting that water-mediated proton transfer initiates the reactions and promotes the binding of C and O atoms.

#### 2.2.3. Intramolecular Proton Transfer Reaction of Anti-CH_3_CHOO with the OSO_3_H Group of GAS

**Figure 6:**
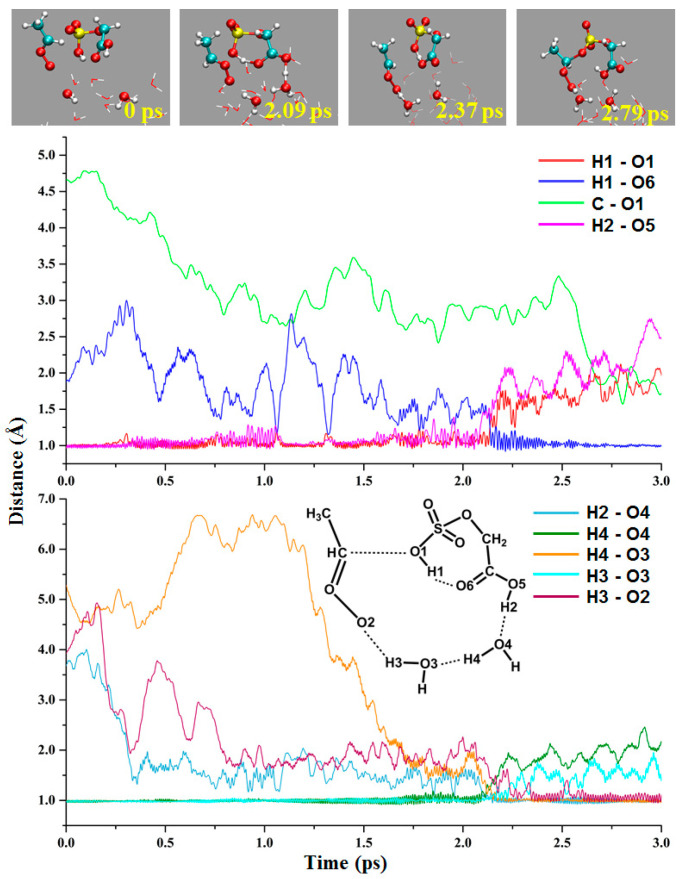
The intramolecular proton transfer reaction between *anti*-CH_3_CHOO and OSO_3_H group of GAS at gas–liquid interface.

Most of the previous studies have focused on the reaction of CIs with monofunctional species [35,36,37]. Even the study of CIs and multifunctional species is the independent reaction of a single functional group [38]. However, the reaction involving both functional groups of GAS is observed in this study (Figure 6). At 0 ps, the two interface water molecules are far away from *anti*-CH_3_CHOO and GAS, and the distances of H2-O4 and H3-O2 are 3.68 and 3.92 Å, respectively. The transition-state-like complex of *anti*-CH_3_CHOO, GAS, and water molecules is formed at 2.09 ps. The proton of the OSO_3_H group is transferred to the carbonyl oxygen of the COOH group at this time; the distances of H1-O1 and H1-O6 are 1.15 and 1.44 Å, respectively. The proton of the COOH group moves to the water molecule, and the distances of H2-O5 and H2-O4 are 1.26 and 1.28 Å, respectively. In this way, proton transfer occurs between the two functional groups of GAS, and the COOH group acts as the shuttle of proton transfer in this reaction. At 2.37 ps, the H3-O2 bond (the length is 0.99 Å) is formed, indicating proton transfer between the four reactant molecules is complete. The C-O1 bond is formed at 2.79 ps, resulting in the formation of new products. During the whole reaction process, both the COOH group of GAS and water molecules act as bridges of proton transfer.

#### 2.2.4. GAS-Mediated Hydration of Anti-CH_3_CHOO

**Figure 7:**
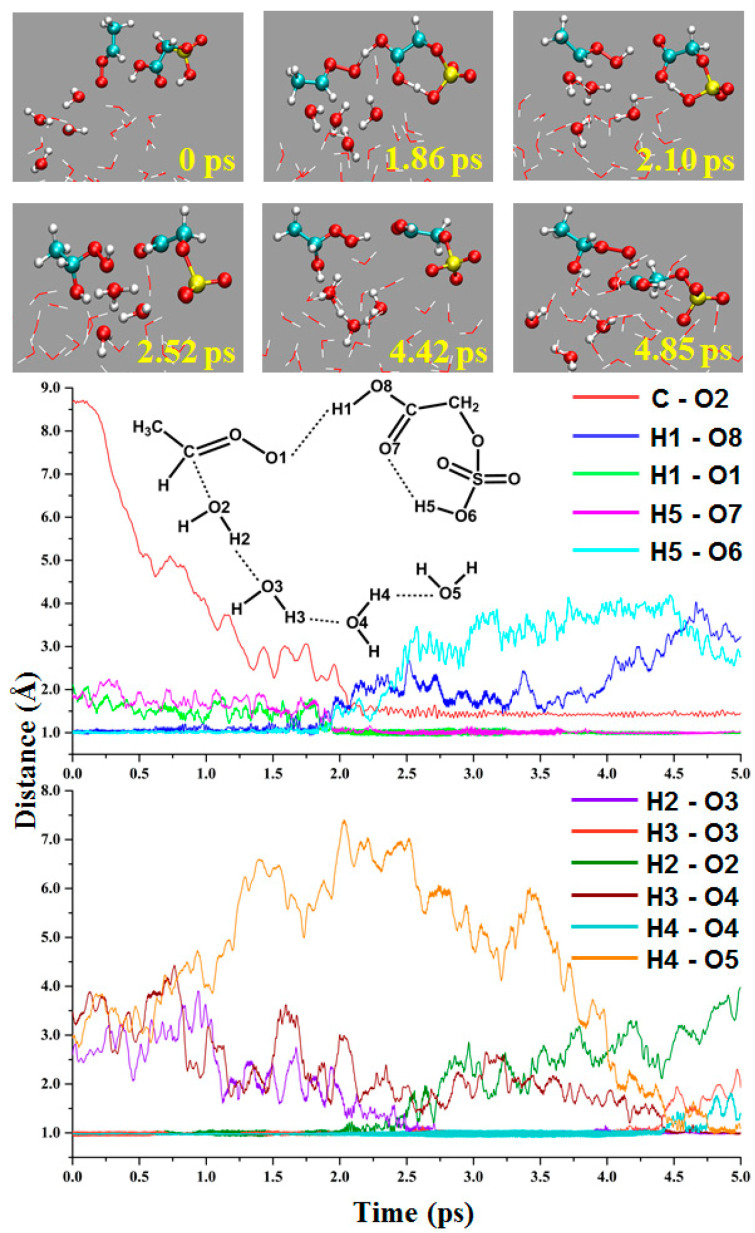
The GAS-mediated hydration of *anti*-CH_3_CHOO at gas–liquid interface.

Kumar et al. [39] reported BOMD simulations of *anti*-CH_3_CHOO reacting with HNO_3_ at the gas–liquid interface, suggesting that HNO_3_-mediated *anti*-CH_3_CHOO hydration is the most dominant reaction. In this study, the GAS-mediated hydration of *anti*-CH_3_CHOO occurs and generates C_2_H_3_O_6_S^-^ and H_3_O^+^ ions, and the reaction follows an obvious stepwise mechanism. As shown in Figure 7, *anti*-CH_3_CHOO and GAS are placed on the droplet surface, which is far from the water molecules at 0 ps. For the first step, the proton on the COOH group is transferred to the terminal oxygen of *anti*-CH_3_CHOO, and the intramolecular proton transfer occurs in GAS. At 1.86 ps, the distances of H1-O1 and H5-O7 are 1.18 and 1.24 Å, respectively. At 2.10 ps, the distances of H1-O1 and H5-O7 are 0.98 and 1.01 Å, respectively, indicating the protonated *anti*-CH_3_CHOO and C_2_H_3_O_6_S^ȡ^ ion are formed. In the second step, the O atom of the water molecule combines with α-C of the *anti*-CH_3_CHOO, and the proton is transferred to another water molecule. At 2.52 ps, the distances of C-O2 and H2-O3 are 1.41 and 0.98 Å, respectively. In the last step, the water molecules transfer protons to each other, and the H_3_O^+^ ion is formed at 4.85 ps. In this pathway, GAS acts as a water molecule to provide protons for the hydration of *anti*-CH_3_CHOO.

## 3. Atmospheric Implications

These results deepen the understanding of CI fate in the atmosphere. GAS is an abundant nucleation precursor and has an important contribution to the formation of atmospheric particulate pollution [40,41,42]. The reaction with GAS is one of the sink pathways for CH_3_CHOO, especially the direct reaction with the COOH group is a nearly barrierless process (1.00 kcal/mol), which may be the dominant reaction in a dry atmosphere. The water-mediated reaction between CH_3_CHOO and OSO_3_H groups is also a nearly barrierless process (0.78 kcal/mol), which may be the dominant reaction in polluted areas with high humidity.

Furthermore, the BOMD simulations reveal the reaction mechanism of *anti*-CH_3_CHOO with GAS on droplets. Both direct and water-mediated reactions are observed, with the formation of ring structures during the reaction. Among them, the water-mediated reaction follows the proton transfer mechanism, and the water molecule acts as a bridge for proton transfer. Although it has been reported that CH_3_CHOO can exist stably on the droplet surface [30], all the reactions of *anti*-CH_3_CHOO with GAS on the droplet occur on the ps timescale. The terminal O atom of *anti*-CH_3_CHOO binding to the H atom of GAS in the reaction occurs slightly later than the α-C atom of *anti*-CH_3_CHOO binding to the O atom of GAS, suggesting the reactions are initiated by the binding of O and H atoms. Both quantum chemical calculations and molecular simulations indicate that water molecules can participate in the reaction of CH_3_CHOO with GAS. In addition, though the reaction of CIs with various species on droplets is discussed in the literature [43,44,45,46], intramolecular proton transfer was observed in our simulations. The hydrogen bond and six-membered ring structure are formed inside the GAS molecule, resulting in the intramolecular proton transfer. It is believed that similar reactions can occur in some substances with longer carbon chains and multiple functional groups in the atmosphere. GAS-promoted hydration of *anti*-CH_3_CHOO as a proton donor was also observed in the simulations. Previous studies have pointed out that nitric acid and methanesulfonic acid promote CIs hydration [39,47]; GAS also tends to perform intramolecular proton transfer while donating a proton, which has appeared many times in the simulations.

The product of CH_3_CHOO reacting with GAS is hydroperoxides. Hydroperoxides participate in the formation of new particles, and SOA in the atmosphere [48,49,50], which have toxic effects on human health and plants [51,52,53], and some macromolecular hydroperoxides can act as CCN [39]. The reactions of CH_3_CHOO with GAS increase the carbon chain length and the ability to form hydrogen bonds, which has great potential in particle formation.

## 4. Materials and Methods

The 72 configurations of GAS were optimized, and the most stable configuration was selected as the reactant. The configuration optimization and transition state searching were conducted with the M06-2X method [54] in conjunction with the 6-311++G(2d,2p) basis set. The analysis of intrinsic reaction coordinates (IRC) [55,56] was used to verify that all transition states connect to the expected reactants and products. Single point energies of reactants, complexes, transition states, and products were calculated at the CCSD/6-311++G(2d,2p) level. All calculations of electronic structures were performed with Gaussian 09 program [57].

Thirty gas–liquid interface simulations were carried out by BOMD. All simulations were performed by CP2K [58] software based on the DFT method. The droplet consisted of 30 water molecules, and reactants were placed in a cubic box with the side length set as 35 Å. The Becke–Lee–Yang–Parr (BLYP) [59,60] functional, double-ζ Gaussian basis set (DZVP) [61] and Goedecker–Teter–Hutter (GTH) norm-conserved pseudopotentials [62,63] were adopted to handle electronic exchange interaction, valence and core electrons, respectively. All BOMD simulations were performed in the constant volume and temperature (NVT) ensemble with the integration step of 1 fs. The system temperature (300 K) was controlled using the Nose-Hoover chain method.

## Data Availability

The configurations of the direct and water-mediated reaction stages (reaction complex, transition state and product).

## Supplementary Materials

The following supporting information can be downloaded at: https://www.mdpi.com/article/10.3390/ijms24043355/s1.
