# Supplementary material for: Theoretical Study on the Gas Phase and Gas–Liquid Interface Reaction Mechanism of Criegee Intermediates with Glycolic Acid Sulfate
Source: Int J Mol Sci. 2023 Feb 8;24(4):3355. doi: 10.3390/ijms24043355 (PMC9965808; doi:10.3390/ijms24043355)
Supplement: Supplementary file 1 [file ijms-24-03355-s001.zip › ijms-2141559-supplementary.pdf]

Supplementary Material for

Theoretical Study on the Gas Phase and Gas-Liquid Interface Reaction Mechanism of Criegee Intermediates with Glycolic Acid Sulfate

Lei Li, Qingzhu Zhang*, Yuanyuan Wei, Qiao Wang, Wenxing Wang

Environment Research Institute, Shandong University,

Qingdao 266237, P. R. China

Keywords: Criegee intermediates, Glycolic acid sulfate, Gas-phase reaction, Aqueous-surface reaction, Proton transfer

---

*Corresponding authors. E-mail: zqz@sdu.edu.cn

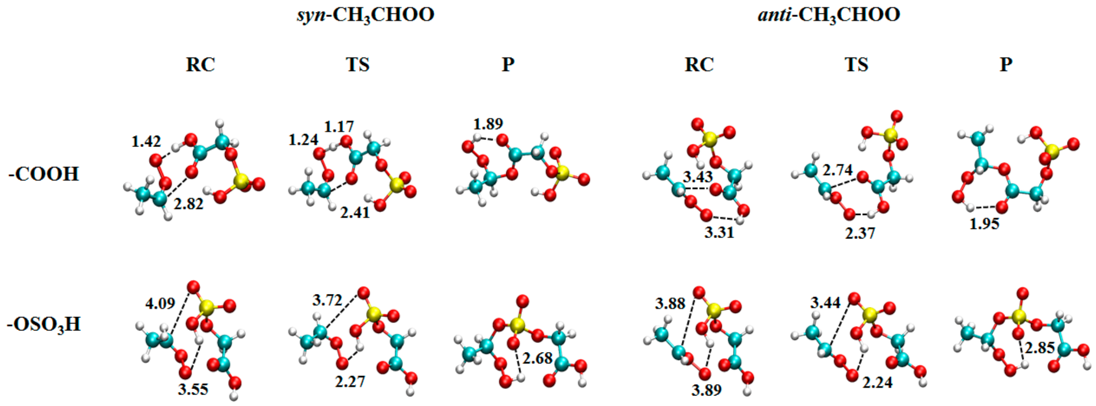

Figure. S1. The configurations of the direct reaction stages (reaction complex, transition state, product).

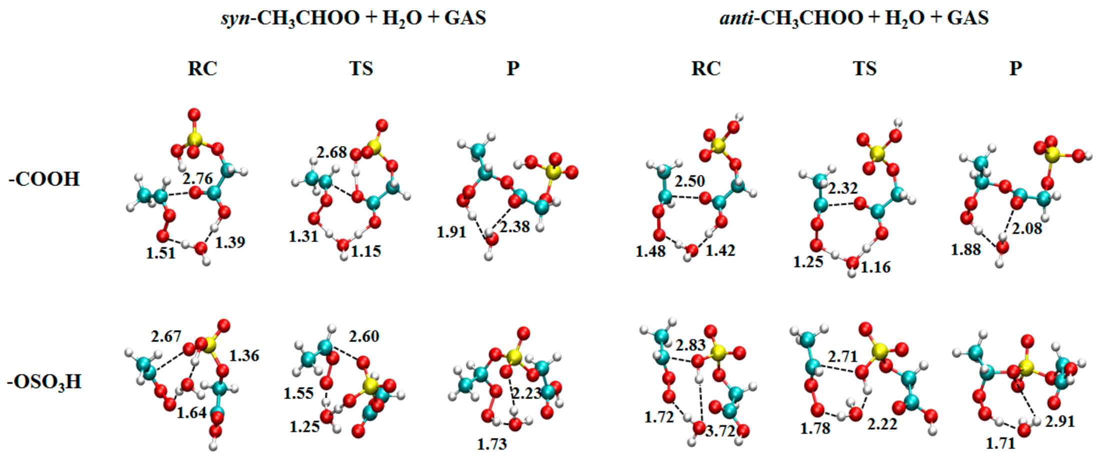

Figure. S2. The configurations of the direct and water-mediated reaction stages (reaction complex, transition state, product).
